# Participant Characteristics Associated With Loneliness Reduction During a Digital Social Robot Intervention Among Older Adults Living Alone in Japan: Secondary Analysis of a Randomized Controlled Trial

**DOI:** 10.2196/105549

**Published:** 2026-10-16

**Authors:** Hiroshi Murayama, Mai Takase

**Affiliations:** 1 Research Team for Social Participation and Healthy Aging, Tokyo Metropolitan Institute for Geriatrics and Gerontology, 35-2 Sakae-cho, Itabashi-ku, Tokyo, 173-0015, Japan, 81 3-3964-3241

**Keywords:** digital social robots, loneliness, social isolation, social contact, older adults living alone, secondary analysis, randomized controlled trial

## Abstract

Among community-dwelling older adults living alone, less frequent preintervention social contact was associated with greater reductions in loneliness during a social robot intervention. Future trials should examine whether preintervention social contact modifies the effects of social robot interventions.

## Introduction

Loneliness is a growing public health concern in aging societies and is associated with higher risks of morbidity and mortality [1]. Psychosocial interventions can reduce loneliness [2]; among these, information and communication technologies, including social robots, have attracted attention as scalable options [3,4]. In a previous randomized controlled trial (RCT), we found that a 4-week intervention using an operator-mediated humanoid communication robot that enabled conversations with operators and family members reduced loneliness and improved psychological well-being among community-dwelling older adults living alone in Japan [5].

Notably, previous studies have primarily reported average intervention effects [4], which may mask variation in individual responses. Existing subgroup analyses have focused on intervention characteristics [2,4]. Thus, little is known about which participant characteristics are associated with greater changes in loneliness during social robot interventions. Identifying such characteristics may generate hypotheses for future trials examining how social robot interventions could be better targeted under resource-constrained scenarios.

In this secondary analysis of our previously published RCT [5], we used intervention-phase data to explore which participant characteristics were associated with greater reductions in loneliness during the intervention. We explored which participant characteristics were associated with greater reductions in loneliness during the intervention, hypothesizing that less frequent preintervention social contact would be associated with larger decreases in loneliness.

## Methods

### Study Design

We analyzed data from an RCT of community-dwelling adults aged 65 years or older who lived alone in Tokyo and neighboring areas and reported loneliness. Participants were randomly assigned to immediate (n=36) or delayed (n=37) intervention sequences. The immediate sequence received the intervention during the randomized trial period, whereas the delayed sequence received the same intervention after the initial control period. Two participants in the immediate sequence dropped out (1 declined participation and 1 had a hearing problem), whereas 4 participants in the delayed sequence declined participation, including 1 during the subsequent intervention period. Thus, preintervention and postintervention loneliness data were available for 67 participants (34 immediate and 33 delayed). Data from both sequences were pooled, with intervention sequence included as a covariate. The intervention was a 4-week program using an operator-mediated humanoid robot (BOCCO emo; Yukai Engineering Inc.) (Figure 1) that provided around-the-clock personalized voice communication. Details of the design can be found elsewhere [5].

**Figure 1.**
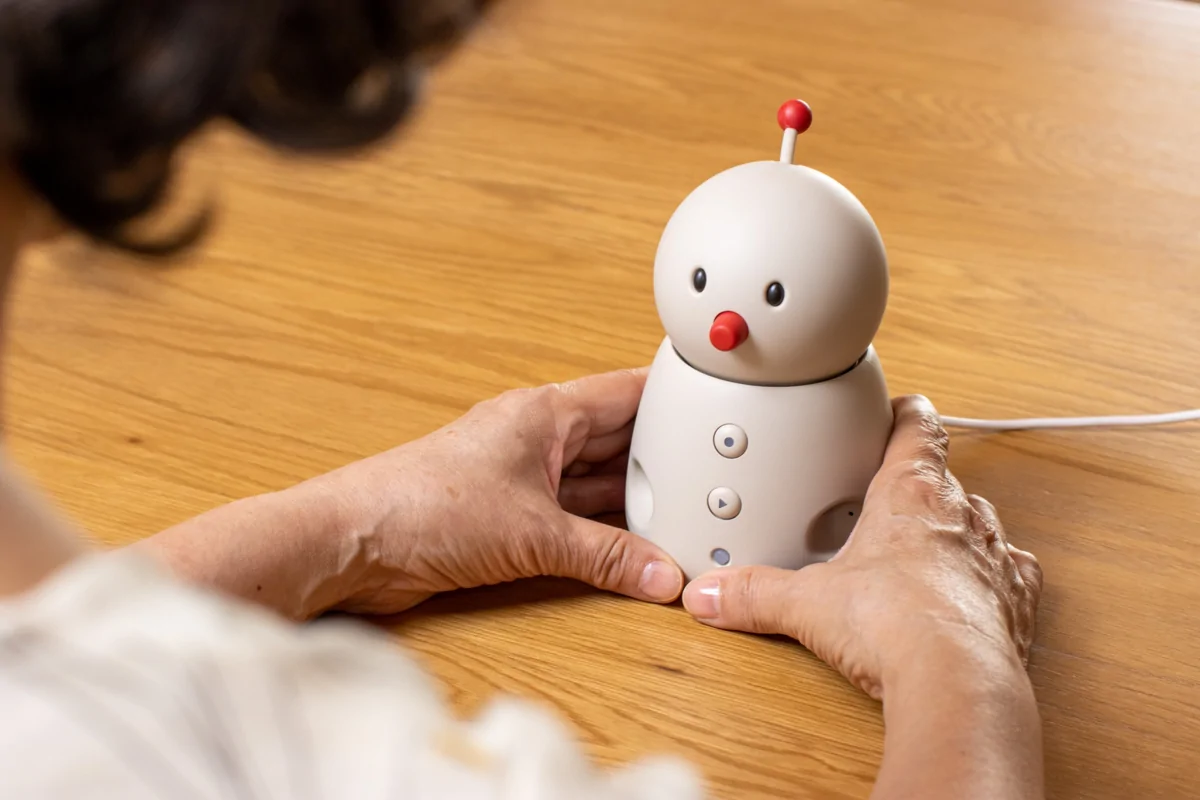
Representative images of BOCCO emo.

The outcome was the difference in the 20-item UCLA Loneliness Scale (version 3 [6,7]; range 20‐80) before and after the intervention, with negative values indicating decreased loneliness. We entered preintervention social contact and loneliness, together with sex, age, marital status, education, subjective financial stability, self-rated health, long-term care certification, and intervention sequence (immediate or delayed), into a single regression model. Frequency of social contact was assessed using 4 items pertaining to face-to-face and remote contact with noncohabiting family members or relatives and with friends or neighbors. The scores were summed and classified as 0 (≥4 times/week), 1 (1‐3 times/week), or 2 (<1 time/week). Preintervention loneliness was dichotomized at the median for the analysis. We fitted multiple linear regression models without (model 1) and with (model 2) an interaction term between social contact and loneliness using IBM SPSS Statistics 29.0 (IBM Corp).

### Ethical Considerations

This study was approved by the Ethics Committee of the Tokyo Metropolitan Institute for Geriatrics and Gerontology (R22-009) and was registered with the UMIN Clinical Trials Registry (UMIN000050644). All participants provided written informed consent. Participants received no financial or other compensation for participation.

## Results

Of the 67 participants, 94% (n=64) were female, and the mean age was 82.3 (SD 6.5) years. Loneliness decreased by a mean of 2.6 (SD 6.4, range −18 to 13) points.

Table 1 presents the factors related to improvements in loneliness. In model 1, less frequent social contact was associated with a greater reduction in loneliness (b −2.14 per category toward less frequent contact, 95% CI −4.31 to −0.04), as was high preintervention loneliness (b −2.52, 95% CI −5.11 to −0.07). Participants assigned to the immediate sequence exhibited less improvement than those assigned to the delayed sequence (b 3.16, 95% CI 0.47-5.86). No other characteristic was associated with changes in loneliness. In model 2, the interaction between less frequent social contact and high loneliness was not statistically significant (b –0.02, 95% CI –5.30 to 5.25).

**Table 1.** Characteristics associated with loneliness improvement following the social robot intervention.

Characteristics	Category	Model 1		Model 2	
		b (95% CI)	*P* value	b (95% CI)	*P* value
Intervention sequence (Ref: delayed)	Immediate	3.16 (0.47 to 5.86)	.02	3.16 (0.44 to 5.89)	.02
Sex (Ref: male)	Female	−2.05 (−7.92 to 3.83)	.48	−2.04 (−8.09 to 4.00)	.50
Age	(Older)	−0.02 (−0.22 to 0.18)	.82	−0.02 (−0.23 to 0.18)	.82
Marital status (Ref: unmarried)	Married	−0.40 (−5.68 to 4.88)	.88	−0.40 (−5.73 to 4.94)	.88
Educational level	(Lower)	−0.76 (−2.12 to 0.61)	.27	−0.76 (−2.14 to 0.62)	.28
Subjective financial stability	(Poorer)	1.35 (−0.51 to 3.22)	.15	1.36 (−0.55 to 3.26)	.16
Self-rated health	(Poorer)	0.34 (−1.23 to 1.92)	.67	0.34 (−1.26 to 1.94)	.67
Long-term care certification	Certified	0.23 (−2.44 to 2.90)	.86	0.23 (−2.50 to 2.95)	.87
Preintervention frequency of social contact	(Less frequent)	−2.14 (−4.31 to −0.04)	.04	−2.13 (−4.81 to 0.56)	.12
Preintervention loneliness (Ref: low [below median])	High (above median)	−2.52 (−5.11 to −0.07)	.04	−2.52 (−5.47 to 0.44)	.09
Preintervention frequency of social contact×preintervention loneliness	—^a^	—	—	−0.02 (−5.30 to 5.25)	.99

a Not applicable.

## Discussion

To our knowledge, this is the first study to examine which participant characteristics were associated with greater reductions in loneliness during a social robot intervention. In model 1, less frequent social contact and higher loneliness before the intervention were each associated with greater reductions in loneliness. The association with less frequent social contact may reflect greater unmet needs for interaction and conversation among participants with little social contact. Social isolation and subjective loneliness are related but distinct [8], and the interaction between them was not statistically significant, although the wide CI renders this finding inconclusive.

Likewise, higher preintervention loneliness was associated with greater reductions in loneliness. This finding should be interpreted with caution. As preintervention loneliness was both a predictor in the model and the initial value used to calculate the change-score outcome, the observed association may partly reflect regression to the mean [9] and mathematical coupling between preintervention loneliness and the change score. We also pooled the intervention-phase data rather than comparing the on-robot and off-robot periods for the same person; therefore, we cannot attribute this association to the robot. Accordingly, the association with preintervention loneliness should be regarded as exploratory.

Overall, these findings should be viewed as hypothesis-generating rather than as evidence of differential treatment benefit. In particular, preintervention social contact warrants testing as a potential effect modifier in future trials.

This study has several limitations. The sample was small, predominantly female, very old, drawn from 1 region, and restricted to individuals who reported loneliness; therefore, the findings may not be generalizable. Moreover, the analysis was exploratory, included multiple predictors, and was not corrected for multiple testing. It identified characteristics associated with loneliness reduction rather than treatment-effect modification. The small sample also limited the power to detect an interaction between preintervention social contact and preintervention loneliness. The smaller reduction observed in the immediate sequence remains difficult to explain but may reflect period-related or other contextual differences because the intervention phases occurred during different study periods for the 2 sequences.

In conclusion, less frequent preintervention social contact was associated with greater reductions in loneliness during the social robot intervention. The association with higher preintervention loneliness should be interpreted cautiously. Future trials should examine whether social contact modifies treatment effects.

## Abbreviations

RCT: randomized controlled trial
